# Characterization of phylogenetic networks with NetTest

**DOI:** 10.1186/1471-2105-11-268

**Published:** 2010-05-20

**Authors:** Miguel Arenas, Mateus Patricio, David Posada, Gabriel Valiente

**Affiliations:** 1 Department of Biochemistry, Genetics and Immunology, University of Vigo, E-36310 Vigo, Spain; 2 Algorithms, Bioinformatics, Complexity and Formal Methods Research Group, Technical University of Catalonia, E-08034 Barcelona, Spain

## Abstract

**Background:**

Typical evolutionary events like recombination, hybridization or gene transfer make necessary the use of phylogenetic networks to properly depict the evolution of DNA and protein sequences. Although several theoretical classes have been proposed to characterize these networks, they make stringent assumptions that will likely not be met by the evolutionary process. We have recently shown that the complexity of simulated networks is a function of the population recombination rate, and that at moderate and large recombination rates the resulting networks cannot be categorized. However, we do not know whether these results extend to networks estimated from real data.

**Results:**

We introduce a web server for the categorization of explicit phylogenetic networks, including the most relevant theoretical classes developed so far. Using this tool, we analyzed statistical parsimony phylogenetic networks estimated from ~5,000 DNA alignments, obtained from the NCBI PopSet and Polymorphix databases. The level of characterization was correlated to nucleotide diversity, and a high proportion of the networks derived from these data sets could be formally characterized.

**Conclusions:**

We have developed a public web server, *NetTest *(freely available from the software section at http://darwin.uvigo.es), to formally characterize the complexity of phylogenetic networks. Using NetTest we found that most statistical parsimony networks estimated with the program TCS could be assigned to a known network class. The level of network characterization was correlated to nucleotide diversity and dependent upon the intra/interspecific levels, although no significant differences were detected among genes. More research on the properties of phylogenetic networks is clearly needed.

## Background

The evolution of DNA or protein sequences that have been the subject of reticulating events like recombination, gene transfer, or hybridization cannot be depicted with a single phylogenetic tree [1-3]. On the contrary, phylogenetic networks allow reticulations among branches rather than imposing a strictly bifurcating structure, and therefore are much more suitable for this task [4-6]. There are different types of phylogenetic networks [5]. Among these, *split networks *(implicit representation) are obtained as a combinatorial generalization of phylogenetic trees, and are designed to represent incompatibilities within and between data sets. In order to be able to accommodate incompatible splits, they contain internal nodes that do not represent ancestral genes or sequences. On the other hand, *reticulate networks *(explicit representation) represent evolutionary histories in the presence of reticulate events such as hybridization, horizontal gene transfer, or recombination, and every internal node represents an inferred ancestor. Most often, phylogenetic networks estimated from real data are depicted as unrooted, especially at the intraspecific level.

Research on phylogenetic networks is just starting, especially if we compare it with all the work that has been done on bifurcating trees, which, indeed, are much simpler structures. This means that many of the calculations that can be easily implemented for bifurcating trees are not available for phylogenetic networks. For example, typically researchers are interested in contrasting different biological hypotheses through the comparison of alternative evolutionary histories. In the absence of reticulation a variety of appropriate metrics exist to measure the distance between two phylogenetic trees (see [7-10]), but in the case of networks this issue is much more complicated.

"Perfect" -that is, that obey non-negativity, separation, symmetry, and triangle inequality-comparison metrics for networks have already been proposed [11-18], together with recognition algorithms that allow for the classification of networks into specific categories. Indeed, the formal characterization of phylogenetic networks is important if we want to compare reticulate hypotheses within a sound framework. For example, a web server http://dmi.uib.es/~gcardona/BioInfo/alignment.php was recently developed by G. Cardona to compare and align explicit phylogenetic networks based on the μ-distance metric [19]. The problem is that from the evolutionary point of view, these categories imply stringent assumptions. We have shown that population genetic models like the coalescent with recombination can result into uncharacterizable phylogenetic networks [20]. However, it is unknown whether this is also the case for networks estimated from real data.

### Definitions

A network contains *nodes *(vertices) and *branches *(edges) connecting them. Here we will refer to *rooted networks *(rooted directed acyclic graphs) with a temporal reference that allows for the identification of *parent *and *child *(descendant) nodes. *Internal nodes *have one or two children while *external nodes *(leaves) have none. The oldest node is called the *root *and has no parents. *Tree nodes *and *hybrid nodes *have one and two parents, respectively. Internal tree nodes have two children, while hybrid nodes have only one child. Nodes that share the same parent are *siblings*. Networks can be classified as *tree sibling *[12,13], where every hybrid node has at least one sibling that is a tree node; *tree child *[11,13,14], in which every internal node has at least one child that is a tree node; *galled-trees *[15,16,21], where the paths from the most recent common ancestor (MRCA) of the parents of a hybrid node down to the hybrid node form disjoint cycles; and (binary) *trees*, which only contain tree nodes. These network classes are nested: tree-sibling ⊃ tree-child ⊃ galled-trees ⊃ trees, meaning a tree is also a galled-tree, a tree-child network, and a tree-sibling network, and so on. Networks that cannot be included in any of these categories are considered *uncharacterizable*. There is also a nested classification of networks as level-k networks [22], where a level-0 network is a tree, a level-1 network is a galled-tree, and, in general, a network is a level-k network if every biconnected component has at most k hybrid nodes. Perfect metrics have also been proposed for level-k networks [23].

## Implementation

### Implementation of the NetTest server

The NetTest web server is composed of an HTML front-page in which the user can upload or paste the network in different formats (see Results). The analysis pipeline was developed in Perl using CGI (Common Gateway Interface). The network classification uses the BioPerl module Bio::PhyloNetwork [13]. The web server uses the Apache HTTP server and was tested and verified using Firefox, Safari, and Internet Explorer.

### Characterization of Reticulate Networks from Empirical Data

**Figure 1:**
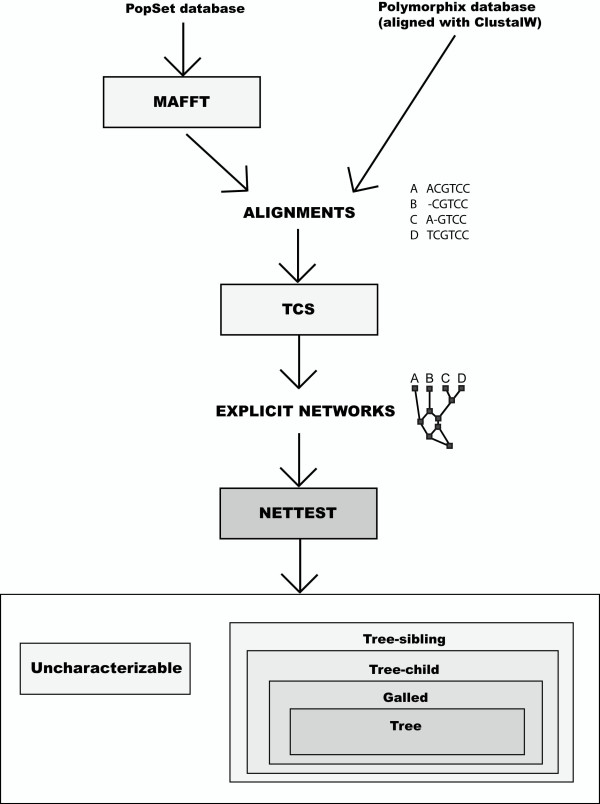
**Characterization of networks from real data sets**. Steps for the characterization of empirical networks: Data sets are aligned and fed to TCS in order to get a reticulate phylogenetic network, which can be input to NetTest for its characterization.

We used *NetTest *to analyze a large group of nucleotide alignments from the *PopSet *database at the NCBI http://www.ncbi.nlm.nih.gov/sites/entrez?db=popset (Figure 1). We downloaded all available data sets for four genes: POL (viral polymerase), COX1 (mitochondrial cytochrome oxidase I), 18S ribosomal RNA (small eukaryotic ribosomal subunit), and ITS1 (ribosomal internal transcribed spacer). In total, we analyzed 565, 1407, 2294, and 191 data sets, respectively, each containing between 9 and 50 sequences. All data sets were aligned using MAFFT [24]. In addition, we also gathered 516 alignments from the Polymorphix database [25], representing a variety of nuclear genes from *Homo sapiens*. This database uses ClustalW [26] to generate the alignments.

All the alignments were fed into the TCS program [27] for the estimation of statistical parsimony networks [28] under the default 95% connectivity limit, which is the maximum number of mutational connections between pairs of sequences justified by the "parsimony" criterion [28]. Note that the presence of divergent sequences can result in the generation of several TCS (sub)networks, in which case were analyzed independently. In a few cases (<1%) the program crashed before completing the analysis, and the corresponding data sets were excluded from the analyses. The resulting networks were rooted according to the node with the highest outgroup weight (i.e., the rooting probabilities described in [29]).

## Results

### NetTest: A web server for the characterization of explicit networks

We have developed a web server called *NetTest*, available from the software section at http://darwin.uvigo.es, able to classify phylogenetic networks into several formal categories (tree, galled-tree, tree-child, and tree-sibling). As far as we know, no other similar programs or servers implement this analysis. All the submitted networks have to be rooted (directed), but they can be specified in different formats:

• Directed GML: The Graph Modelling Language is the standard file format in the Graphlet graph editor system (see http://www.infosun.fim.uni-passau.de/Graphlet/GML/).

• TCS-GML: The program TCS [27] can generate unrooted networks in GML format (saved as *.*graph *files). In this case, the network is automatically directed by NetTest assuming that the "root" is the node with the highest outgroup weight. If the file submitted to the server contains several networks, *NetTest *analyzes them in a sequential fashion.

• NEXUS-SplitsTree: The program SplitsTree [30] can produce rooted networks using the "rooted equal angle algorithm" that can be exported in NEXUS format (*.nex) and feeded directly to NetTest.

• Extended Newick (eNewick) [19]: This is the type of representation used for the directed networks simulated with Netgen [31] or estimated with Phylonet [32]. The leftmost occurrence of each hybrid node in an eNewick string corresponds to the full description of the subnetwork rooted at that node.

• DIMACS standard [33]: This is a widely used format for representing graphs, developed for the DIMACS Challenge http://dimacs.rutgers.edu/Challenges/. NetTest will consider that all edges are directed and that the first node of each edge is the parent node.

• Branch list: A simple list of connections *node x *to *node y*, where *x *corresponds to the parent node and *y *to the descendant node.

The main output of NetTest is a depiction of the network and an indication of whether it can be characterized under the tree, galled-tree, tree-child, and tree-sibling categories. The results of the analysis can also be downloaded. NetTest also implements a feedback for questions, requests, and bug reports, and a help page is included with detailed information.

### Network classes resulting from empirical data

**Figure 2:**
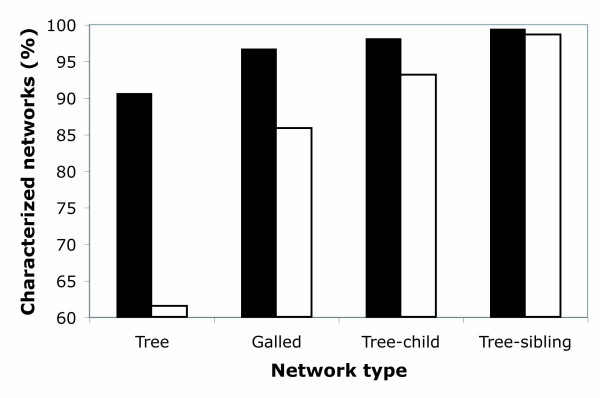
**Network complexity and intra/interspecific level**. Characterization of TCS networks derived from the PopSet data sets according to their intraspecific (black bars) and interspecific (white bars) composition.

**Figure 3:**
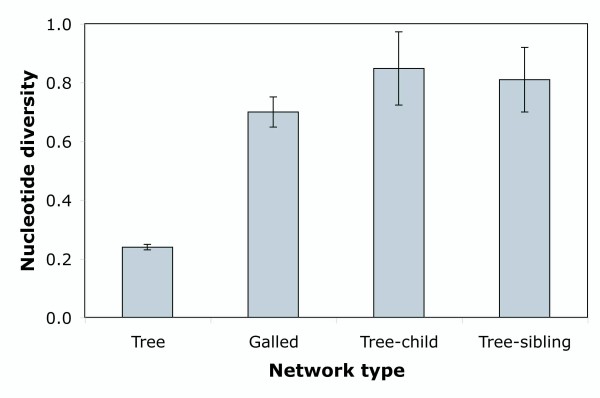
**Network complexity and diversity**. Characterization of TCS networks derived from the PopSet data sets according to nucleotide diversity. Error bars indicate 95% confidence intervals.

Using NetTest we found that most networks (98.5-99.6%) could be characterized regardless of the gene analyzed (Table 1). The analysis of the human data sets also resulted in a high number of characterizable networks (Table 1). For PopSet data sets, those networks containing a majority of sequences corresponding to the same species (*intraspecific *data sets) resulted in a significantly higher percentage (χ^2 ^*P*-value < 2.2e^-16^) of characterization for any class assignment (especially for tree, galled-tree, and tree-child) than networks derived from data sets where several sequences belonged to more than one species (*interspecific *data sets) (see also Figure 2). Also, the level of characterization depended on nucleotide diversity (ANOVA *P*-value < 2 × 10^-16^). In particular, data sets resulting in trees were significantly less diverse than data sets resulting in more complex networks (Figure 3).

**Table 1:** Characterization of TCS networks.

Nature of data sets	POL	COX1	18S	ITS1	*Human*
Number of data sets	566	1417	2303	191	516

Number of networks generated by TCS	1535	3889	6041	531	1035

Hybrid nodes	495 0.32	891 0.23	914 0.15	117 0.22	124 0.12

Tree	1326 (86.4%) [0 0.00]	3428 (88.1%) [0 0.00]	5505 (91.1%) [0 0.00]	465 (87.6%) [0 0.00]	952 (92.0%) [0 0.00]

Galled-tree	1443 (117) (94.0%) [131 1.11]	3738 (310) (96.1%) [348 1.12]	5873 (368) (97.2%) [410 1.11]	507 (42) (95.5%) [53 1.26]	1015 (63) (98.1%) [67 1.06]

Tree-child	1495 (52) (97.4%) [161 3.10]	3799 (61) (97.7%) [169 2.77]	5941 (68) (98.3%) [179 2.63]	518 (11) (97.6%) [27 2.45]	1022 (7) (98.7%) [16 2.29]

Tree-sibling	1523 (28) (99.2%) [103 3.68]	3865 (66) (99.4%) [241 3.65]	6019 (78) (99.6%) [232 2.97]	523 (5) (98.5%) [11 2.20]	1033 (11) (99.8%) [36 3.27]

Uncharacterizable	12 (0.8%) [100 8.33]	24 (0.6%) [133 5.54]	22 (0.4%) [93 4.23]	8 (1.5%) [26 3.25]	2 (0.2%) [5 2.50]

For each gene, the table indicates the total number of data sets studied (both databases), the total number of networks generated by TCS, the total number and mean (per network) of reticulate nodes and the number of networks classified as tree, galled-tree, tree-child, and tree-sibling. In parenthesis we indicate the number of networks in that category that did not belong to the previous category and the percentage of characterized networks. In brackets we indicated the number and mean (per network) of reticulate nodes. The last row indicates the number of uncharacterizable networks (i.e., more complex than tree-sibling).

## Discussion and Conclusions

NetTest is able to analyze rooted phylogenetic networks in several formats and assign them to different network classes -those for which perfect metrics exist. The application is useful not only for theoretical studies, for example in the design of new metrics, but also for measuring the complexity of the evolutionary processes in real or simulated data sets.

Using NetTest we have shown that most of the TCS networks resulting from the analysis of real data can be classified as tree-sibling, tree-child, or galled-tree networks. Indeed, this result is dependent on the fact that TCS was designed for the analysis of closely related sequences. The TCS connection limit forces complex networks to break apart into simpler ones, therefore favouring their assignment to known network categories. This also explains why the TCS networks are simpler than the ancestral recombination graphs simulated with the coalescent [20].

Appropriate metrics exist for the comparison of empirical characterizable networks (see for example [13]) like those produced by TCS, allowing us to contrast reticulate hypotheses in a formal fashion. Only a small fraction of the TCS networks estimated here were more convoluted than any of these classes, suggesting that more research is needed if we want to compare and analyze more complex phylogenetic networks estimated from real data.

## Availability and requirements

Project name: NetTest

Project home: http://darwin.uvigo.es, software section

Operating system(s): Platform independent

Programming language: Perl with BioPerl library

Requirements: None

Licence: GNU GPL

Any restrictions to use by non-academics: None

## Authors' contributions

MA and GV carried out the network analyses. MP and MA gathered the data sets and MP developed the web server. DP conceived and coordinated the study. All authors participated in its design and helped to draft the manuscript. All authors read and approved the final manuscript.
